# Current treatment of biliary Sphincter of Oddi Dysfunction

**DOI:** 10.3389/fmed.2024.1380640

**Published:** 2024-05-17

**Authors:** Hong-Ze Zeng, Hang Yi, Song He, Rong Wu, Bo Ning

**Affiliations:** Department of Gastroenterology, The Second Affiliated Hospital of Chongqing Medical University, Chongqing, China

**Keywords:** Sphincter of Oddi Dysfunction, endoscopic sphincterotomy, Sphincter of Oddi manometry, EPISOD, Rome IV

## Abstract

The sphincter of Oddi is a delicate neuromuscular structure located at the junction of the biliary-pancreatic system and the duodenum. Sphincter of Oddi Dysfunction (SOD) can result in various clinical manifestations, including biliary-type pain and recurrent idiopathic pancreatitis. The management of SOD has been challenging. With the publication of the landmark Evaluating Predictors and Interventions in Sphincter of Oddi Dysfunction (EPISOD) trial and the Rome IV consensus, our clinical practice in the treatment of SOD has changed significantly in recent years. Currently, the management of type II SOD remains controversial and there is a lack of non-invasive therapy options, particularly for patients not responding to endoscopic treatment. In this mini review, we aimed to discuss the current knowledge on the treatment of biliary SOD.

## Introduction

The sphincter of Oddi (SO) is a delicate neuromuscular structure located at the junction of the biliary-pancreatic system and the duodenum, regulating the flow of bile and pancreatic juice. Sphincter of Oddi Dysfunction (SOD) is a benign motility disorder of the biliary and/or pancreatic sphincter, associated with biliary-type pain and recurrent idiopathic pancreatitis. SOD is often diagnosed in patients with a history of cholecystectomy and has been considered an important cause of post-cholecystectomy syndrome (1, 2). According to the modified Milwaukee classification system, biliary SOD is classified into 3 types based on symptoms, biochemical abnormalities, and imaging results (3, 4). Type I SOD is defined as biliary-type pain with both elevated liver enzymes and a dilated bile duct. Type II SOD presents with biliary-type pain with either elevated liver enzymes or a dilated bile duct. Type III SOD patients have biliary-type pain only without biochemical or imaging abnormalities. This classification has been widely used in clinical practice, as the therapeutic response to sphincterotomy appeared to vary by the type of SOD (1). Type I SOD is now recognized as being related to anatomical stenosis rather than a functional disorder, and the term “SO stenosis” is proposed (1). Traditionally, manometry-directed sphincterotomy was indicated for type II and type III SOD (3). However, the landmark Evaluating Predictors and Interventions in Sphincter of Oddi Dysfunction (EPISOD) trial indicated no benefit of sphincterotomy for patients with type III SOD (5). In the Rome IV consensus, type III SOD was abandoned as a diagnosis and considered as a functional pain, while type II SOD was recommended to be termed suspected functional biliary sphincter disorder (1). Since then, our clinical practice in the treatment of SOD has undergone significant changes. This mini review is aimed to focus on the current knowledge on the treatment of biliary SOD. Given the ongoing use of the Milwaukee classification by many clinicians, we have decided to maintain the original nomenclature in this review.

## Endoscopic therapy

Endoscopic sphincterotomy (EST) is the most commonly used non-pharmacologic treatment for type I and type II biliary SOD (6). For type I SOD patients, sphincterotomy without manometry has been recommended, as these patients generally benefit from sphincterotomy regardless of the results of Sphincter of Oddi manometry (SOM) (1, 3). Rolny et al. (7) investigated 17 post-cholecystectomy patients categorized to biliary type I SOD who underwent EST or surgical sphincterotomy (7). Despite 6 patients displaying normal SOM results, all reported symptom relief during a mean follow-up of 28 months, suggesting that SOM in type I SOD may be unnecessary and potentially misleading (7). Similarly, a study by Sugawa et al. (8) examined the effect of EST without SOM in 8 type I SOD patients, with all participants achieving symptom resolution (8).

The treatment of type II SOD is more controversial. Two early randomized sham-controlled trials indicated that EST was more effective in type II patients with elevated basal sphincter pressures (Table 1) (9, 10). In the study by Geenen et al. (9), forty-seven post-cholecystectomy patients with biliary-type pain meeting type II SOD criteria were randomly assigned to receive either EST or a sham procedure, with prior SOM performed on all participants. At the 1-year follow-up, 10 of 11 patients with elevated basal pressures who underwent EST showed significant improvement in symptoms and remained asymptomatic at the 4-year follow-up. By comparison, only 3 of 12 patients showed clinical improvement in the sham group. Seven patients who did not improve with the sham procedure at 1 year subsequently received sphincterotomy and all of them were free of symptoms at the 4-year follow-up. Among the patients with normal basal pressures, symptom relief was comparable between those who underwent sphincterotomy and those in the sham group, suggesting that sphincterotomy provided no significant benefit in type II SOD patients with normal sphincter pressure (9). Another randomized sham-controlled clinical trial by Toouli et al. (10) showed similar results. In this study, SOM was performed in 81 patients with biliary-type pain after cholecystectomy who had a dilated bile duct and/or increases in liver enzymes. Among patients with elevated basal sphincter pressure, symptoms improved in 11 of 13 treated by sphincterotomy and in 5 of 13 assigned to a sham procedure. Relief of symptoms was significantly more common in patients treated by sphincterotomy than in those having a sham procedure at 24 months. In contrast, patients with sphincter dyskinesia or normal SOM did not benefit more from sphincterotomy than sham procedures.

**TABLE 1:** Randomized controlled trials including type II biliary SOD.

		Geenen et al. (9)	Toouli et al. (10)
**Design**		**Sham-controlled**	**Sham-controlled**
No. of patients		47	81^#^
**Treatment success**			
Elevated SOM	EST	10/11 (91%)	11/13 (85%)
	Sham	3/12 (25%)	5/13 (38%)
Non-elevated SOM	EST	5/12 (42%)	12/24 (50%)
	Sham	4/12 (33%)	13/29 (45%)
**Complications**			
Pancreatitis		2	7
Perforation		1	1
Bleeding		1	–

SOD, Sphincter of Oddi; SOM, Sphincter of Oddi manometry; EST, Endoscopic sphincterotomy.

^#^The authors did not provide specific information about the classification of SOD. However, according to the numbers of participants with various entry criteria, this trial mainly included type II SOD with small numbers of type I and type III. Two patients were excluded from analysis due to lost to follow-up or withdrawal from the study.

Despite previous recommendations of SOM-guided EST for type II SOD, manometry is not widely available and the management of this type of SOD varies in different institutions and among clinical practitioners. A survey of U.S. expert endoscopists revealed that SOM is not routinely performed and many endoscopists have reservations concerning its invasive character and reliability (11). A recent study demonstrated poor reproducibility of SOM and further questioned its clinical value (12). Empirical biliary sphincterotomy performed by experienced endoscopists without manometry has shown to provide comparable clinical results and may be more cost-effective in comparison to a SOM-guided strategy (13–15). Consequently, this approach has become increasingly common for type II SOD patients (1, 15). It is of note that many endoscopists believe type II SOD is at least in part a functional disorder with limited response to sphincterotomy and thus may turn to other approaches instead (11). In recent years, endoscopic injection of botulinum toxin (BTX) into SO has demonstrated positive results in SOD patients with minimal adverse events (16–19). However, the effect of BTX has been shown to be transient and there is some concern that repetitive injections may cause fibrosis of the SO (19, 20). Nonetheless, positive response to BTX may have a predictive value in identifying patients who might benefit from sphincterotomy, offering a safer alternative to SOM (17–19, 21, 22). Despite these developments, robust randomized controlled trials on sphincterotomy for type II SOD remain scarce. Many studies have been retrospective, unblinded, conducted with small sample sizes, and have not used objective assessments (1). Clinical trials with high evidence levels are needed.

Previous studies showed that the efficacy of sphincterotomy in biliary type III SOD patients with abnormal manometry varied widely, ranging from 0 to 56%, and the validity of sphincterotomy in these cases has long been questioned (5, 23). To assess the effectiveness of sphincterotomy and the predictive value of SOM, a multicenter, sham-controlled, randomized trial (the EPISOD trial) was conducted (5). This trial involved 214 patients who had post-cholecystectomy pain but presented no significant abnormalities on imaging or laboratory studies, and had no history of pancreatitis or prior sphincter intervention. Patients were randomized in a 2:1 ratio to sphincterotomy or a sham procedure irrespective of manometry findings. Those assigned to sphincterotomy with elevated pancreatic sphincter pressures were further randomized in a 1:1 ratio to biliary or dual (biliary and pancreatic) sphincterotomy. Success of treatment was defined as a reduction in their Recurrent Abdominal Pain Intensity and Disability (RAPID) score with no narcotic use and no further sphincter intervention. The rate of successful outcome at 12 months was 23% for sphincterotomy and 37% for sham treatment, with no significant difference between biliary sphincterotomy (20%) and dual sphincterotomy (30%) in patients with pancreatic sphincter hypertension. Furthermore, manometry results were not predictive of the outcomes. Comparable findings were observed in the EPISOD 2 study, which included 72 patients who declined randomization and underwent manometry-directed sphincterotomy (24). The long-term outcomes of the EPISOD trial at up to 5 years demonstrated similar results and further confirmed that sphincterotomy is no more effective than sham intervention for type III SOD and should not be performed in these patients (24). On the basis of EPISOD, type III SOD was removed from the Rome IV consensus and there has been an immediate and sustained decrease in the utilization of sphincterotomy for newly diagnosed SOD (25). Additionally, whether type III SOD should be considered a distinct condition has been debated and some studies have suggested other causes including psychosomatic disorders, central sensitization, and visceral hyperalgesia (26–30).

## Medical therapy

While EST can be effective for type I and type II SOD, endoscopic retrograde cholangiopancreatography (ERCP) carries risks of adverse events. Notably, SOD is one of the primary risk factors for post-ERCP pancreatitis and ERCP-related perforation (31). Therefore, it is crucial to explore conservative therapies, and medical treatments should be attempted prior to invasive procedures (1, 32).

There have been limited trials of pharmacologic treatments for SOD. The main agents adopted so far include calcium channel blockers, antispasmodics, and nitrates, which relax the SO and modulate its basal pressure. Two short-term placebo-controlled cross-over trials showed that nifedipine, a calcium channel blocker, effectively reduced pain in SOD patients (33, 34). However, a subsequent study revealed that long-term slow-release nifedipine offered no advantage over placebo and was associated with frequent side effects (35). Trimebutine, an opioid agonist, regulates SO motility based on the basal SO motility anomalies in patients with post-cholecystectomy pain (36). In one study, a combined treatment of trimebutine and a nitrate derivative was as effective as sphincterotomy, with only 23% of the patients resorting to endoscopic therapy (37).

Antidepressants are occasionally prescribed to reduce visceral hypersensitivity in patients with irritable bowel syndrome. Similarly, this approach has been applied to SOD treatment. In one study, 21 of 59 patients obtained symptom resolution or improvement when treated with low-dose tricyclic antidepressants (mainly amitriptyline) in combination with nitrates or analgesics (14). The efficacy of duloxetine, a serotonin-norepinephrine reuptake inhibitor, was indicated in an open-label, single-center clinical trial but adverse events limited its use (38).

To date, medical therapy for SOD has been disappointing with inconsistent outcomes. Although certain strategies have demonstrated potential in the treatment of SOD, most studies were uncontrolled with a limited level of evidence considering the high placebo response in functional disorders (1).

## Surgical therapy

Before the development of endoscopic techniques, SOD was primarily managed by surgical treatment. Previous studies have demonstrated good outcomes of transduodenal sphincteroplasty (TS) (39–41). However, in the current era of therapeutic endoscopy, endoscopic interventions are now the preferred treatment option, with surgical therapy reserved for carefully selected patients (1, 39–41). A retrospective review by Morgan et al. (39) was conducted to evaluate the efficacy of TS for SOD and pancreas divisum. This study showed that individuals with a history of gastrointestinal bypass surgery generally had more favorable outcomes, while younger age and chronic pancreatitis appeared to predict poorer results (39). Another retrospective study by Schwartz et al. (41) which analyzed patients undergoing either endoscopic or surgical therapy for SOD after gastric bypass, revealed that treatment success and duration of remission was higher in those treated by surgery with similar morbidity and mortality.

## Conclusion

The management of SOD presents significant challenges. The publication of EPISOD and the Rome IV consensus has profoundly changed our clinical practice in the treatment of SOD, particularly for type III patients. Currently, the management of type II SOD remains controversial and there is a notable deficiency in non-invasive therapy options, especially for patients who do not respond to endoscopic interventions. There is a critical need for randomized controlled clinical trials that provide high-level evidence with unified classifications and standardized metrics for evaluation.

## Author contributions

H-ZZ: Writing – original draft, Writing – review and editing. HY: Writing – review and editing. SH: Writing – review and editing. RW: Writing – review and editing. BN: Conceptualization, Data curation, Supervision, Writing – review and editing.
